# Interviews with Irish healthcare workers from different disciplines about palliative care for people with Parkinson’s disease: a definite role but uncertainty around terminology and timing

**DOI:** 10.1186/s12904-016-0087-6

**Published:** 2016-02-09

**Authors:** Siobhan Fox, Alison Cashell, W. George Kernohan, Marie Lynch, Ciara McGlade, Tony O’Brien, Sean S. O’Sullivan, Suzanne Timmons

**Affiliations:** Centre for Gerontology and Rehabilitation, St. Finbarr’s Hospital, Douglas Road, Cork City, Ireland; Parkinson’s Association of Ireland, Dublin, Ireland; Institute of Nursing and Health Research, University of Ulster, Co., Antrim, UK; Irish Hospice Foundation, Dublin, Ireland; Centre for Gerontology and Rehabilitation, School of Medicine, University College Cork, Cork, Ireland; Marymount University Hospital and Hospice, Co., Cork, Ireland; Cork University Hospital, Cork, Ireland

**Keywords:** Parkinson disease, Parkinsonism, Palliative care, Interviews, Integrated care model, Qualitative research

## Abstract

**Background:**

An integrated palliative care approach is recommended in all life-limiting diseases, including Parkinson’s disease (PD). However research shows that people with PD have unmet palliative care needs. The study aimed to explore multidisciplinary healthcare workers’ (HCWs) views on palliative care for people with PD, identifying perceived barriers and facilitators.

**Methods:**

A qualitative design was used; data was analysed using Thematic Analysis. Semi-structured interviews were conducted with 30 HCWs, working either with people with PD or in a palliative care setting in Ireland.

**Results:**

A number of perceived barriers were evident helping to account for the previously reported unmet palliative care needs in PD. A lack of education about PD and palliative care meant that HCWs were unsure of the appropriateness of referral, and patients and carers weren’t equipped with information to seek palliative care. A lack of communication between PD and palliative care specialists was seen to impede collaboration between the disciplines. Uncertainty about the timing of palliative care meant that it was often not introduced until a crisis point, despite the recognised need for early planning due to increased prevalence of dementia.

**Conclusions:**

Most HCWs recognised a need for palliative care for people with PD; however several barriers to implementing a palliative care approach in this population need to be addressed. Implications for clinical practice and policy include the need for an integrated model of care, and education for all HCWs, patients, carers, and the public on both the nature of advanced PD, and the potential of palliative care in support of patients and their family members.

## Background

Palliative care is “an approach to care that improves the quality-of-life of patients and their families facing problems associated with life-threatening illness, through the prevention and relief of suffering by means of early identification and impeccable assessment and treatment of pain and other problems, physical, psychosocial and spiritual”[1]. Palliative care is applicable to all people with a life-limiting illness, for the duration of their illness. Parkinson’s disease (PD) is a life-limiting neurodegenerative disorder that is currently incurable and thus all treatment is symptomatic.

People with PD may benefit from i) generalist palliative care approach which involves all healthcare workers (HCW) practicing palliative care principles as a core skill, supplemented by some HCW who are not engaged full time in palliative care, but have had additional training and experience in palliative care, perhaps to diploma level; ii) Specialist Palliative Care (SPC) services whose core activity is the provision of palliative care to individuals with more complex and demanding care needs [2].

It is now widely accepted that people with PD and their carers would benefit from a palliative care approach, with episodic input from SPC. An exemplary Specialist Palliative Neurology service in the UK found better outcomes for people with neurological illness (mainly PD or PD plus syndromes), using the service [3]; for example, mean hospital admissions were 0.9 % per patient in the last year of life and only 26 % died in hospital, compared with national averages of 3.5 % and 46 %. However outside of these exceptional centres, very few people with PD receive palliative care, or are referred for SPC input, compared to those with malignant disease [4].

It must also be recognised that there are differences in how palliative care may apply to PD rather than to other illnesses such as cancer. For example there is a very long duration of disease, a fluctuating and unpredictable disease course, complex specialist treatments, high prevalence of cognitive impairment, and people with PD often die ‘with’ not ‘from’ their illness [5].

Richfield conducted a review of PD and palliative care needs, including the small qualitative literature [6]. These studies mainly consisted of interviews with family carers. Recurrent themes include the following: the emotional impact of diagnosis, changing social roles, financial difficulties and the carer strain which results when a family member develops PD. Participants also describe a lack of information around the time of diagnosis and difficulty requesting information from healthcare workers. Furthermore, Hasson et al. [7], studying the end-of-life experience of family members, described a lack of preparedness for the death of loved ones and poor knowledge of SPC services.

Many HCW feel the palliative care needs of people with PD are currently unmet [8], reflective of previously reported views of carers of people with PD [7, 9]. Some previous qualitative research has examined barriers to palliative care in PD from the perspectives of HCW working in Northern Ireland. Focus groups and interviews with 12 Allied Health Professionals (AHPs) identified PD palliative care as being impeded by several barriers [10]: personal (e.g. limited knowledge and experience), organisational and administrative (e.g. poor rate and timing of referrals), and perceptual (e.g. perceived negative perceptions of palliative care among PD patients). A subsequent study using mainly focus-groups with 13 social workers [11] found they had varying interpretations and sometimes negative associations of palliative care. Other barriers included insufficient knowledge and experience of PD and a lack of resources.

Further research is needed to examine why people with PD in the Republic of Ireland may still have unmet palliative care needs, despite Irish policy and research recommendations [12, 13]. To expand on previous work with AHPs, an exploration of the potential barriers from the perspectives of a range of HCW is needed, including multiple disciplines who work in PD or palliative care and, critically, including consultant physicians, the group most likely to make referrals to SPC. Others have argued that in order to encourage the development of new services, which address this inequality in palliative care access, we need to understand the nature of palliative care needs in PD [6], including all key stakeholders. This study directly addresses this gap through a qualitative exploration of HCW from multiple disciplines’ views on palliative care in PD; the central research question being: what are the main barriers and facilitators to providing palliative care (generalist or specialist) to people with PD?

## Methods

### Research team

A project Steering Group was established to oversee a research programme of palliative care needs in PD, which includes this study. The Steering Group consists of seven PD and palliative care experts, including academic and clinical researchers, and representatives from PD and palliative care advocacy groups.

The first author (S.F.) conducted all of the interviews and the analysis, with expert input from the Steering Group. S.F. is a female researcher with a PhD in psychology and has experience of conducting quantitative and qualitative research in Parkinson’s disease.

### Design

The current study employed a qualitative, exploratory, inductive research design, using one-to-one in-depth interviews. This methodological approach was chosen to help achieve a greater understanding of the issues identified by our previous survey [8]; in-depth semi-structured interviews allow for follow-up and probing of issues discussed, and the emergence of pertinent issues that may not be pre-defined in the interview schedule. Thus, a semi-structured interview schedule was developed, based on a previously published interview study in PD and palliative care [10], and modified following discussion with the Steering Group. A pilot interview was conducted, and minor changes to the schedule were made. The key topic areas of the final interview schedule can be seen in Table 1. Demographic information was recorded using a simple questionnaire.

**Table 1:** Interview schedule

Palliative care:	
1.	How would you define palliative care?
2.	Do you think a patient with Parkinson’s disease has palliative care needs?
Perceptions of patients’ and carers’ palliative care needs	
3.	What do you feel are the main palliative care needs of a patient with Parkinson’s disease?
4.	What do you feel are the main palliative care needs of carers and families of a patient with Parkinson’s disease?
Delivery of palliative Care	
5.	When do you think is the most appropriate time to deliver palliative care to a patient with PD?
6.	When do you think is the most appropriate time to begin talking about palliative care with a patient and/or their carer and family?
7.	Who do you think should initiate the conversation about palliative care? Why?
8.	Would you feel comfortable talking about palliative care with a patient with PD?
Experiences of services:	
9.	What specific palliative care interventions do you think people with PD could benefit from?
10.	What specific palliative care interventions do you think carers and families of people with PD could benefit from?
11.	What type of palliative care interventions (if any) do you provide/Do you refer patients to?
Barriers and facilitators to delivering palliative care:	
12.	Can you tell me about any barriers that exist which might hinder your ability to deliver palliative care to people with PD?
13.	Can you tell me about any barriers that exist which hinder your ability to deliver palliative care to families/carers of patients with PD?
14.	What factors would help you to facilitate the delivery of palliative care?
15.	What barriers (if any) exist to providing integrated palliative care services?
16.	Have you any suggestions on how a joint care approach might be implemented?
Education and training	
17.	What topics would you like to see included on a training module for palliative care for patients with PD?
Additional comments	
18.	Is there anything else that you would like to add that you did not get a chance to discuss?

### Ethics

This study received ethical approval from the Clinical Research Ethics Committee of the Cork Teaching Hospitals.

### Participants

HCWs were recruited using a purposive sampling strategy, i.e. people initially known to the project Steering Group to be actively working in one of the areas of PD or palliative care. Palliative care is not routinely associated with PD in Ireland, thus the inclusion criterion was that participants must be working/expert in either of these disciplines, but not necessarily both. Additional HCW in acute or community settings in the Health Service Executive South region in Ireland were then recruited via limited snowball sampling. None were acquainted with the interviewer. Exclusion criteria were HCW not involved in the care of people with PD or palliative care; HCW based in a region other than HSE south; HCW still in training (e.g. student placement).

Participants were contacted personally via email or telephone by S.F. who was not known to any of the participants prior to this study; this limited a personal influence in recruiting participants. All participants received a study information sheet and provided written and oral informed consent to participation.

Thirty HCW were recruited to allow representation of a range of disciplines and career stages; recruitment ceased then as data saturation was achieved.

### Data collection and analytic strategy

The first author (S.F.) conducted all interviews and the analysis, to ensure familiarisation and a more faithful interpretation of data. Most interviews lasted 30-50 minutes, taking place in a private room in the participant’s workplace. Interviews were audio-recorded (with permission), transcribed, anonymised, and then analysed using Thematic Analysis [14]. The data was analysed using NViVo software version 10, which assisted in storage, searching and coding of the large amount of data obtained. To help identify and minimise potential bias, a reflexivity diary was logged throughout the data collection and analysis phases.

During transcription, initial ideas were noted in memos, as outlined by Riessman [15]. Transcripts were re-read and recordings listened to several times, to ensure transcription accuracy and to increase immersion in the data. Data was coded to identify features pertinent to the central research question. For quality assurance purposes, a second researcher reviewed a random sample of 20 % of the transcripts and the resulting data coding. The next stage involved searching for themes; to explain larger sections of the data by combining similar or linked codes. ‘Thematic maps’ [14] were used to visualise data and consider relationships between themes. Finally, the themes were named and defined, and exemplary quotes were selected which best reflected the meaning of each theme. Data were analysed and reported according to Consolidated Criteria for Reporting Qualitative Research (COREQ) guidelines wherever possible [16].

## Results

All 30 HCWs approached agreed to participate in the interviews. Participants’ demographic details, clinical roles, and work locations are detailed in Table 2.

**Table 2:** Demographics of participants (N = 30 healthcare workers) who gave interviews.

Sex	*n*
Male	*4*
Female	*26*
Setting	
Acute Hospital	*17*
Day Hospital	*7*
Long term care	*4*
Community	*1*
Hospice	*1*
Title	
Staff nurse	*6*
Clinical Nurse Manager	*5*
Consultant Geriatrician	*3*
Consultant Neurologist	*3*
Clinical Nurse Specialist palliative care	*2*
Senior Physiotherapist Care of Elderly	*2*
Consultant Palliative Medicine	*1*
Consultant Physician	*1*
Registrar (Neurology ward)	*1*
Clinical Nurse Specialist neurology	*1*
Clinical Nurse Specialist elderly care	*1*
Advanced Nurse Practitioner Rehab Older Adult	*1*
Senior Speech and Language Therapist	*1*
Senior Occupational Therapist	*1*
Practice Development Facilitator	*1*

Notes: The average time in their current position was eight years (range 0.5 to 15 years). Participants reported seeing an average of 16 people with PD per month (average range 1 to 100)

Thematic Analysis of the data revealed two over-arching themes, and eight subthemes, namely:

- The role of palliative care in PD
  - o Palliative care needs in PD
  - o The enigma of PD palliative care needs
  - o Knowing when to introduce palliative care in PD
  - o Is general or specialist palliative care more appropriate in PD?
- Barriers to palliative care in PD
  - o Education for HCW, patients and families, and the public
  - o Healthcare Workers’ personal barriers
  - o Stigma of palliative care
  - o Practical barriers

## The role of palliative care in PD

### Palliative care needs in PD

HCWs varied in their definitions of palliative care, ranging from “*care in the last few hours”* or *“preparation for death”* to *“care of people whose disease is no longer curative”.* Many focused on quality-of-life, comfort care, and holistic care. One HCW highlighted the range of interpretations of the term ‘palliative care’.

*“My first question to you is what are you calling palliative care? That’s my thing is everybody calls it something different.”* Interview 30, Senior Speech and Language Therapist

Although few HCWs had significant experience with palliative care in PD, most felt that people with PD would benefit from palliative care, and that palliative care should be available to anyone who needs it, regardless of diagnosis. HCWs with experience of palliative care in PD valued it highly.

The support provided by SPC was seen as most useful in advanced PD, for conditions such as aspiration pneumonia, and swallowing difficulties. SPC was also seen as useful in an advisory/consultancy role for complex cases involving ethical dilemmas or complex discharges. SPC professionals recognised their role in providing person-centred care; including the carer and family; supporting spiritual and emotional well-being; and providing home care support.

*“I suppose in XX they get seen for about 10-15 minutes, you know there’s a plan and off they go. Whereas from a palliative care point of view, we give time. We link in daily.”* Interview 21 Clinical Nurse Specialist in Palliative care

Some non-specialists found it difficult to think of symptoms requiring SPC, tending to only associate palliative care with pain management.

### The enigma of PD palliative care needs

HCWs identified ways in which a palliative care approach may be different in PD than in other illnesses. They highlighted that PD is a complex disease that can present with different symptoms, and can progress gradually or sometimes quite rapidly towards advanced stages. Thus, one person may have very different needs to the next.

However many more HCWs, typically those some palliative care training or experience, felt that the palliative care needs of people with advanced PD are similar to anyone else seeking palliative care towards the end of life, and that managing symptoms is the core of any palliative care approach, regardless of diagnosis

*“From my point of view now looking after someone with COPD, someone with a stroke, someone with end stage renal failure, they all have similar symptoms near the end, you know their breathing could be laboured, you know pain-wise, so you know you assess all those areas and you treat them and you give them effective symptom control for all those areas that they need.”* Interview 21, Clinical Nurse Specialist in Palliative Care

Palliative care specialists recognised that people with advanced PD may actually be more accepting of palliative interventions than people with other diseases, having lived with their condition for a long time.

*“[People with PD have] travelled that journey …. their family has coped with it over the years and seen their loved one[‘s] condition deteriorate gradually and it’s come to a point where they just want their loved one to be comfortable and I suppose any help or any teams that can help are welcome.”* Interview 21, Clinical Nurse Specialist in Palliative Care

Often PD won’t be the direct cause of the person’s death, they might get an acute illness such as pneumonia and die quite suddenly. HCWs highlighted that it is better for the patient and family to be clear from the outset that PD is incurable, and to have realistic expectations. This will help them to cope with advancing disease and deterioration, and facilitates acceptance of palliative care.

### Knowing when to introduce palliative care in PD

This theme was marked by uncertainty among participants. HCWs feel that they are slower to recognise the palliative stage of PD than other neurological illnesses. Often cited was the particular difficulty in defining the end-of-life phase in PD.

*“I suppose the deterioration can be quite slow, you know [in] a PD patient, and it can be quite subtle, that people nearly miss it; it’s very gradual, so it’s quite a hard one”* Interview 12, Clinical Nurse Specialist in Neurology

There was further uncertainty as to the right time to refer a patient with PD to SPC, with the individual nature of this illness often cited. Many different ‘triggers’ were mentioned such as:

*“When you feel that the quality-of-life of the patient is really declining”* Interview 15, Consultant Physician

*“You might be looking at PEG [Percutaneous endoscopic gastrostomy] feeding”* Interview 30, Senior Speech and Language Therapist

*“When you feel like you’re not able to manage the symptoms”.* Interview 3, Consultant Neurologist

HCWs were further divided on when to introduce a person with PD to the concept of palliative care; answers varied from “*at diagnosis”:*

*“perhaps it should be something that we have for the PD patient at diagnosis so we say now this is a whole lot of information, some of it is relevant to you now, some of it isn’t, you know about exercise, diet, driving, planning ahead for the future, and maybe about palliative care.”* Interview 23, Consultant Geriatrician

to not discussing it until the *“advanced stages”, “when they’ve had the disease for more than 10 or 15 years”* or *“when the drugs aren’t working anymore”.* Other HCWs suggested an individualised approach, such as being sensitive to *“cues from the patient*”, allowing the conversation to happen *“naturally”* or at “*an opportunistic time”.* The prevalence of dementia in PD was cited by HCWs as mandating earlier conversations around palliative care and advance care planning.

Further uncertainty concerned whether palliative care input should be available throughout the PD course, or restricted to advanced stages. Some felt that episodic involvement of SPC teams throughout the illness is preferable.

*“palliative care … would come in and just address a particular issue that was causing problems for people and then move back out again, that’s the way I’d see it.”* Interview 28, Senior Occupational Therapist

Uncertainty as to best timing existed, however finding the right timing to introduce these supports was seen as critical; when referred at the right time patients and families welcome palliative care input. HCWs cited various key workers who should first introduce palliative care to a patient; however most agreed that this should be a person who has an established rapport with the patient and family.

### Is general or specialist palliative care more appropriate in PD?

HCWs felt that effective palliative care delivery to people with PD requires up-skilling and learning, with palliative care personnel training in PD management, or as one participant put it, training in *‘neuro-palliation’.* Similarly, PD specialists, and everyone working with PD, require generalist palliative care competencies.

Some argued that many palliative care needs can be met by generalists. Advantages include a seamless service, receiving care in a single setting, and established rapport. Furthermore, there were fears that frequent referral to SPC might de-skill geriatricians or neurologists. However most felt that when patients’ needs are complex, input from SPC is needed.

Many participants felt that the best model was one of integrated care; that PD experts should continue to deal with specialised PD medication, complicated on-off effects, hallucinations, etc., and that they should therefore continue to work alongside their colleagues in SPC. Critically, participants felt that patients with PD should have ready access to both general or SPC throughout their illness, depending on their needs.

*“What [people with PD] should be able to do is cross seamlessly between those settings, so they should be able to access the acute setting if they need it, they should certainly be able to access generalist palliative care at any time, and if their needs are complex they should have a mechanism to access SPC.”* Interview 31, Consultant in Palliative Medicine

## Barriers to palliative care for people with PD

### Education for HCW, patients and families, and the public

Across the interviews, lack of education was identified as a primary barrier. PD was perceived as a complex disease and that specialist training is needed to manage it effectively.

HCWs also felt that further general education and awareness around palliative care is needed, this being a ‘*grey area*’. Most felt that there is still a misperception among most HCWs that palliative care is applicable only in the final days of life.

Education needs for patients and families were another barrier. One HCW felt that educating patients and families about the benefits of palliative care would encourage them to take initiative in contacting these resources:

*“… if you educate people about what’s available to them or what might be available to them, I think that would also go a long way so they would ask, because patients and families asking for something is really powerful, ‘we would like to see the palliative care team’”.* Interview 31, Consultant Palliative Medicine

Some felt that patients and families needed more education on PD symptomology, especially non-motor aspects which may be overlooked, thus empowering patients to seek help for symptoms which mightn’t otherwise be discussed at a routine clinic appointment.

*“And I do think actually the patients with PD often aren’t aware that some of their other symptoms are due to PD. So for example I think that hallucinations..... they don’t seem to realise they should be reporting it or that it’s a significant symptom of PD.”* Interview 23, Consultant Geriatrician

A final theme was that palliative care in non-cancer illnesses may be facilitated through promoting public awareness and tackling stigma at a societal level; *‘we need to educate the public at large that it’s not just about the morphine drip’.*

### Healthcare workers’ personal barriers

One barrier often cited was that non-palliative specialists often are ‘*not comfortable*’ or ‘*afraid’* of talking about palliative care; some actively avoid having these conversations with patients and families.

*“It’s us again is the barrier. You know how comfortable are you to sit down and discuss [palliative care] with them, our palliative care girls are very very good in that area, they’re used to it, ya. But it’s us I think is the barrier being honest. It is us.”* Interview 13, Clinical Nurse Manager Care of the Older Adult

Another barrier identified was that some consultants were reluctant to ‘hand over’ their patients to palliative care, clearly not considering an integrated care model.

*“Some of them see it almost as if ‘I’m handing over care to the palliative care team’, rather than it being a combined [approach].”* Interview 18, Staff Nurse

One consultant neurologist discussed the *‘delusional optimism’* among HCWs that PD is treatable. Another spoke of modern culture promoting medical treatment and searching for a ‘cure’, as opposed to focusing on quality-of-life and symptom management.

*“It’s a difficult call for the consultant to make. I suppose in this day and age it’s all medical treatment and you know I see it here sometimes you wonder when are you going to draw the line and say enough is enough here.”* Interview 21, Clinical Nurse Specialist in Palliative Care

### Stigma of palliative care

All HCWs referred to the negative stigma of palliative care. They said that it is associated with death and cancer in the minds of patients, families, the public, and even many HCWs. Many spoke about the fear associated with the words ‘palliative care’; that HCWs are hesitant to ‘scare’ patients; while the hospice is perceived to be associated with approaching death.

*“I think palliative care in general probably has that stigma attached to it that it’s just for cancer patients who are about to die”* Interview 2, Consultant Neurologist

Participants suggested different methods of tackling this stigma. Some suggested the terminology be changed, such as referring to *‘symptom control’.* Others suggested that once people have contact with a palliative care team, this negative stigma will be expelled. Education is also important to surmount this barrier.

*“No it’s [the stigma] surmountable. Once you do something.... and people see it works … they get confidence in it.”* Interview 17, Consultant Geriatrician

### Practical barriers

These included resource and geographical barriers. Almost all HCWs identified insufficient funding and resources. HCWs spoke of the ‘fight’ for resources, and long waiting lists, which impede the delivery of palliative care in PD, as in other services. Many mentioned staffing barriers, notably a lack of psychologists, social workers, and sometimes AHPs.

However many also pointed out that this resource barrier is universal in health services, and shouldn’t be used as a reason not to seek palliative care involvement for people with PD.

A geographical barrier was often cited. HCWs cited an inequity in palliative care resources across Ireland. There are few PD nurse specialists; in one region where one was available they were described as having an *‘ad hoc’* role. It may be especially difficult for someone with advanced PD to travel even relatively short distances to clinics and services, a problem compounded in rural areas where transport services are poorer and distances to clinics greater.

### Final comments

HCW spoke of how the introduction of palliative care in PD could be facilitated by tackling the identified barriers, i.e. through education and awareness campaigns for HCW, patients/carers, and the public; guidelines to aid referral and timing; increased support for staff who want to attend training in palliative care, and increasing resources where needed.

## Discussion

All HCWs, including doctors, nurses, AHPs, and other care staff, have a role in the delivery of palliative care to people with PD, throughout the illness and especially in advanced disease [17]. This study found that, although most HCWs felt that people with PD could benefit from a palliative care approach and/or input from SPC, many were unsure of when and how these may be appropriate in PD. Very few HCWs, from either the palliative care or PD background, had experience of providing or referring to palliative care for PD, consistent with low referral rates reported in quantitative literature [4, 8].

When defining ‘palliative care’ the confusion between it and end-of-life was evident, although most did identify the holistic and patient-centred features of palliative care.

Several barriers impeding the delivery of palliative care to people with PD were identified. Misconceptions were common, for example some HCWs in this study were unsure how their patients with PD could benefit from palliative care as they “don’t have pain”. However research has shown that pain is the second most common symptom ‘dominating the day’ for people with PD [18]. Furthermore palliative care is applicable for many more symptoms beyond pain management.

Previous interview-based research with patients and carers showed that they often found it difficult to request information and discuss palliative care with HCWs [9, 19]. This is reflected in the current study where HCWs themselves confessed reluctance to discuss palliative care issues, and may be slow to engage with patients/carers. However HCWs also admitted here that the best time to discuss these issues was upon a cue from the patient, in line with previous research in other illnesses [20]. HCW must overcome their personal barriers and become more confident in dealing with palliative issues, so as to not inhibit service development.

Overall, participants showed marked uncertainty about when and how palliative care should be introduced and delivered, and had different views on this. Similar findings have been reported elsewhere [10, 21]. In this study, some suggested earlier exposure would address fear and stigma, however in practice palliative care was not discussed until a crisis point. This is reflective of research in other neurological diseases where such discussions are reactive rather than planned [22]. This is furthermore in line with the results of Richfield et al’s review [6] of the qualitative literature who found that ‘information tension’ was one of the most consistent findings in the literature.

While some negative stigma was attached to palliative care, evident in the fact that many thought that early involvement or discussion of palliative care may scare patients and their families, others highlighted the benefits of early SPC input, including opportunity to build a rapport with the team, to dispel any myths about palliative care early on, and to facilitate a model of episodic input from SPC throughout the patient’s journey. This is in line with current recommendations [23].

Furthermore, earlier SPC involvement is critical because of the increased rates of dementia among people with PD. About 60 % of patients will develop dementia within 12 years of diagnosis [24] and the risk of mild cognitive impairment is double that of the general population even at presentation [25]. This means that early discussions about advance care planning are particularly important for people with PD. While the patient is often met with a great deal of new information at diagnosis, discussions/introductions about palliative care should happen early in the disease trajectory. The current results suggest that HCWs must push themselves to have these discussions, and should be open to cues from the person with PD as to the right time to have these discussions.

As with previous research [11], practical issues were identified; these included a perceived lack of funding, insufficient staffing, already long waiting lists, and geographical barriers such as uneven service distribution across the country. The World Health Organisation has identified neurological disorders as one of the greatest threats to public health [26], and more people are living longer with PD. Therefore palliative care for PD will become a more pressing issue, and appropriate funding must be allocated to SPC services to extend services based on need. However, improving palliative care in PD may not carry a significant financial cost, as many improvements to care can be realised through training existing staff in general palliative care skills.

A key policy issue underlying many themes which emerged in this research was the need for an integrated model of care, which wasn’t identified in past research with single HCW disciplines. Increased communication and collaboration between specialities is important to dispel misconceptions, increase referrals, and enable palliative care professionals to become experienced in caring for people with PD; this is important as past research has shown that many hospice staff are not familiar with neurological illnesses [27]. PD specialists must not view SPC input as ‘handing over care’, but recognise that the combined expertise of both disciplines provides the best overall quality of care for people with PD. Experienced participants in this research suggested that most palliative care needs in people with PD should be provided within existing and developing disease management programmes, with SPC responding where needs become complex and extraordinary. Such integrated approaches are identified as key to providing a high quality of care in the most cost and resource-effective manner [26].

## Conclusion

This study has identified key barriers, and through suggestions to tackle these – facilitators, to delivering palliative care to people with PD. This study adds to the literature by considering the perspectives of several HCW disciplines. Findings indicate that palliative care may be facilitated in this population by increased public awareness of the role of palliative care, education for HCW in both specialist and generalist settings, education for patients and families, better communication and integrated care models, and increased resources. A key clinical implication is that all HCWs need to be trained in the assessment of palliative care needs of their PD patients. The implication for policy is that clear evidence-based guidelines should be introduced to promote the adoption of a palliative care approach, including referral to SPC where needed. This current study, the largest and most comprehensive multiple discipline exploration of HCW views, adds to the previous qualitative literature in this area, together mandating that people with PD should be receiving palliative care from their HCW team. Future research needs to apply specific palliative interventions in this population and use rigorous quantitative and qualitative process and outcome measures, to drive practice change in this important area.

## Availability of data and materials

The dataset is not publicly available as it consists of transcribed interviews which are potentially identifiable, even though names of people and places have been changed.

## Abbreviations

AHP: Allied health professional
HCW: Healthcare worker
PD: Parkinson’s disease
SPC: Specialist palliative care
